# The effect of motorcycle helmet type, components and fixation status on facial injury in Klang Valley, Malaysia: a case control study

**DOI:** 10.1186/1471-227X-14-17

**Published:** 2014-08-03

**Authors:** Roszalina Ramli, Jennifer Oxley, Peter Hillard, Ahmad Farhan Mohd Sadullah, Roderick McClure

**Affiliations:** 1 Department of Oral and Maxillofacial Surgery, Universiti Kebangsaan Malaysia Medical Centre, 56000 Jalan Yaacob Latif, Kuala Lumpur, Malaysia; 2 Monash University Accident Research Centre (MUARC), Monash University Malaysia, Jalan Lagoon Selatan, 46150 Bandar Sunway, Selangor Darul Ehsan, Malaysia; 3 Monash Injury Research Institute (MIRI), Building 70, Monash University, 3800 Monash, VIC, Australia; 4 School of Civil Engineering, Universiti Sains Malaysia, 14300 Nibong Tebal Penang, Malaysia

**Keywords:** Helmet type, Helmet component, Helmet fixation, Facial injury, Klang Valley, Malaysia

## Abstract

**Background:**

The effectiveness of helmets in reducing the risk of severe head injury in motorcyclists who were involved in a crash is well established. There is limited evidence however, regarding the extent to which helmets protect riders from facial injuries. The objective of this study was to determine the effect of helmet type, components and fixation status on the risk of facial injuries among Malaysian motorcyclists.

**Method:**

755 injured motorcyclists were recruited over a 12-month period in 2010–2011 in southern Klang Valley, Malaysia in this case control study. Of the 755 injured motorcyclists, 391participants (51.8%) sustained facial injuries (cases) while 364 (48.2%) participants were without facial injury (control). The outcomes of interest were facial injury and location of facial injury (i.e. upper, middle and lower face injuries). A binary logistic regression was conducted to examine the association between helmet characteristics and the outcomes, taking into account potential confounders such as age, riding position, alcohol and illicit substance use, type of colliding vehicle and type of collision. Helmet fixation was defined as the position of the helmet during the crash whether it was still secured on the head or had been dislodged.

**Results:**

Helmet fixation was shown to have a greater effect on facial injury outcome than helmet type. Increased odds of adverse outcome was observed for the non-fixed helmet compared to the fixed helmet with adjusted odds ratio (AOR) = 2.10 (95% CI 1.41- 3.13) for facial injury; AOR = 6.64 (95% CI 3.71-11.91) for upper face injury; AOR = 5.36 (95% CI 3.05-9.44) for middle face injury; and AOR = 2.00 (95% CI 1.22-3.26) for lower face injury. Motorcyclists with visor damage were shown with AOR = 5.48 (95% CI 1.46-20.57) to have facial injuries compared to those with an undamaged visor.

**Conclusions:**

A helmet of any type that is properly worn and remains fixed on the head throughout a crash will provide some form of protection against facial injury. Visor damage is a significant contributing factor for facial injury. These findings are discussed with reference to implications for policy and initiatives addressing helmet use and wearing behaviors.

## Background

The motorcycle is one of the main modes of transport in Malaysia and constitutes 47% of all registered vehicles in Malaysia [1,2]. Malaysia experiences a high number of motorcycle-related crashes and fatalities, with 120,156 reported crashes and 4,036 deaths in 2010 (In the same year, there were 6,260 fatalities, 6,002 serious injuries and 10,408 light injuries involving all road users] [2]. Despite many interventions addressing motorcyclist safety, this proportion has not decreased over the last decade. The prevalence and severity of head injury and its association with motorcycle helmet use has been well established [3], however, the issues surrounding facial injuries and association with helmet use have received less attention [3,4].

The cranium and the face are closely related, and facial injuries are a frequent outcome of motorcycle collisions. Emotional distress due to aesthetic complications and functional impairment has been recognised as one of the major outcomes of facial injuries. Post traumatic stress disorder following facial injury was shown to be present between 26% and 41% of adults sustaining facial injury [5-7], and long-term consequences are not always directly related to the severity of the injury [8-11].

There are three main factors to consider when addressing the effectiveness of helmets including i) whether or not a helmet is worn, ii) the protective characteristics of different types of helmets, and iii) fixation status. With respect to the first of these, there is overwhelming evidence that wearing a helmet in comparison with not wearing a helmet is beneficial [4,12-16]. The Cochrane review conducted in 2008 concluded that helmets reduced risks of death by 42% and serious head injury by 69% [3]. Given the importance of wearing a helmet, many initiatives have been implemented in the Association of Southeast Asian Nations (ASEAN) countries to increase wearing rates and include legislation, enforcement and education measures, however, the current wearing rate is still very low in some countries [1,17].

Second, is the issue of helmet type and includes the regulation of helmet standards as well as helmet types worn. The standard of all helmets available within the Malaysian market is monitored by the Standards and Industrial Research Institute of Malaysia (SIRIM) which benchmarked the European ECE 22.05 as its gold standard [12]. A range of helmets are available for motorcyclists, and Malaysian motorcyclists generally wear four types of helmets: the half-head, the tropical helmet, the open-face and the full-face, the popular ones being the first two types. Little evidence exists regarding the protective nature of different types of helmets and their components, in relation to either head and/or facial injury.

Third, is the issue of helmet fixation. Helmet fixation is defined as the position of the helmet during the crash whether it was still secured on the head or had been dislodged. A helmet is only effective if it stays on the head during an impact. Helmet dislodgement through improper fastening has the potential to negate the protective features of wearing a helmet. Two improper fastening methods are described in the literature; loosely secured straps and totally unfastened helmets. There is little research to date, however, addressing the effect of poor helmet fastening or securing on head injury. A recent study in Taiwan suggested that wearing a loosely fastened helmet may compromise any potential protection of a helmet [13]. There is also some evidence that poor helmet fastening is relatively prevalent in many Asian countries [14-20], and that there is weak law enforcement in the effort to improve helmet wearing behavior [19-21]. In Malaysia, while there have been policy statements regarding helmet use and proper fitting and securing, reported in the Motorcycles (Safety Helmets) Rules since 1973 [22], there may be a lack of targeted enforcement and education on helmet fixation.

The effect of improper fastening of helmets and fixation of helmet during a crash on the risk of facial injury has not been reported in the research literature. The effect of helmet components, particularly the visor, also remains unknown. The aim of this study was to address these specific gaps in the literature, with view to supporting improved road safety policies and reduce the burden of facial injury in motorcyclists throughout the ASEAN countries.

## Methods

### Study design and setting

The study involved a case control analysis of a series of injured motorcyclists in southern Klang Valley, Malaysia, who presented to hospital for treatment of their injuries or who died from these injuries over a 12-month study period, i.e. from 14 March 2010 to 13 March 2011.

Klang Valley comprises the Federal Territory Kuala Lumpur and part of the State of Selangor and it is located in the Central Region of Peninsular Malaysia. Klang Valley is 2,843 square kilometres in size and has a population close to 6 million people [21,23]. Four major hospitals, one Forensic Institute (National Institute of Forensic Medicine or NIFM) and three Police departments were included in the study.

### Population and participants

The study population was defined as a motorcycle rider or pillion who was involved in a crash within the study region during the study period, and sustained acute injury to any part of the body sufficiently serious that either resulted in death or presentation to hospital for treatment of that injury.

The participant inclusion criteria were: all motorcyclists (rider or pillion); all ethnic groups; all age groups and gender; all types and severity of injuries, and; were involved in a motorcycle crash within the catchment area and within the study period. Motorcyclists who did not sustain any injury, or discharged themselves from hospital care without a definitive diagnosis, and those involved in a road crash outside Klang Valley were excluded. Those who could not comprehend in English or Malay Language or did not consent to participate in this study were also excluded.

The sample of injured motorcyclists was recruited through two sources: police and hospital registries. Fatally injured motorcyclists (those who died instantaneously and brought to the mortuary directly) were identified from the Kuala Lumpur, Ampang and Kajang Police Registries and this list was cross-matched with the selected hospital mortality registries. Injured motorcyclists who attended the selected hospitals and subsequently died within 30 days from date of crash were recruited through the hospital recruitment process.

The cases and controls were described as:

Cases: injured motorcyclists who were recruited through the sources described as the above. These motorcyclists sustained facial injuries with or without other body part injuries.

Controls: injured motorcyclists were also recruited through the same sources as the cases. These motorcyclists sustained all range of injuries except facial injuries.

### Variables and instruments

Data were obtained from three sources; i) participant questionnaires, ii) review of police and medical records and, iii) helmet component laboratory data.

The questionnaire was designed to gather information regarding i) participant demographic characteristics, ii) motorcycle and crash characteristics, and iii) basic riding and helmet wearing and type characteristics.

**Figure 1:**
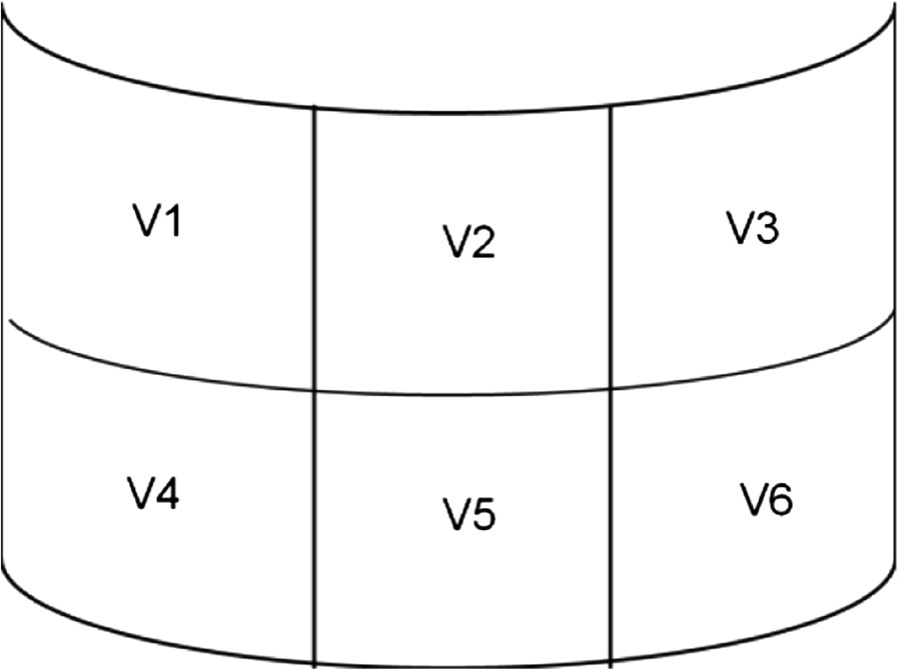
Segment locations for damage description (visor).

Medical and police record data extraction form was used to obtain rider, crash, injury and treatment details. All injury diagnoses were scored and recorded using the Abbreviated Injury Scales (AIS) [24]. The face was defined as an area in front of the head between the ears and from the chin to the hairline [25]. Consistent with clinical practice, the face was categorised into upper, middle and lower face using two horizontal imaginary lines drawn through the inter-pupillary plane and across the commissure of the mouth. These divisions were included as outcomes of interest as some divisions may be more susceptible to injuries than others especially in helmet component damage. The problem of overlapping anatomy between the upper face (described as the area between the hairline to the eye brows) and the frontal region (head injury) was resolved by allocating injuries of the skin and subcutaneous tissue as the upper facial injuries while deeper structures injuries, i.e. bone and brain were grouped under head injury. This method complemented the AIS scoring. For middle and lower face injuries, injuries included those from the superficial to the deepest layer. Police-based data which included police investigation reports (witness interview, vehicle and crash site investigations and crash photographs) were collected from the respected Police Departments and clinical records which included autopsy and toxicology reports were collected from all relevant hospitals and NIFM.Helmet component laboratory analysis was performed on a sample of helmets obtained from injured participants. Helmet component variables selected for laboratory-based analysis included: type of helmet, type of visor, certified visor or otherwise, visor thickness, visor degradation (using the Fourier Transform Infrared (FTIR) spectroscopy) and visor damage. For the assessment of damage, the visor was divided into six segments according to the anatomy of the face where it coincided (Figure 1). The horizontal segments represented the middle (V1 to V3) and lower face (V4 to V6), while the vertical segments represented the right side of face (V1 and V4), the centre of face (V2 and V5) and left side of the face (V3 and V6). The upper face coincided with the visor holder.

### Ethical approval

The study was approved by Monash University Human Research Ethics Committee (MUHREC), Ministry of Health of Malaysia (MOH) and all the selected hospitals and police departments where the participants were recruited. Informed consent was given by all participants and relatives.

### Statistical analysis

The data were analysed using the Predictive Analytics SoftWare (PASW: formerly SPSS) statistics version 18.0 (SPSS Inc., Chicago, IL). Descriptive statistics of the rider, crash and helmet characteristics and injury outcomes of interest were undertaken. Descriptive statistics and simple logistic regression with unadjusted odds ratio were shown in Tables 1 and 2. A kappa coefficient (κ) was determined in the assessment of visor damage where two independent observers were involved.

**Table 1:** Motorcyclist and crash characteristics in southern Klang Valley, Malaysia

**Characteristics**	**Participants, n (%)**			
	**Facial injuries**	**Non-facial injuries**	**Unadjusted OR (95% CI)**	**P-value**
	**(n = 391)**	**(n = 364)**		
**Motorcycle user:**				
Pillion	35 (9.0)	48 (13.2)	Reference	-
Rider	356 (91.0)	316 (86.8)	1.55 (0.97-2.45)	0.07
**Gender:**				
Female	30 (7.7)	43 (11.8)	Reference	-
Male	361 (92.3)	321 (88.2)	1.61 (0.99-2.63)	0.06
**Age (in years):**				
<16	15 (3.8)	26 (7.1)	Reference	-
16-25	191 (48.8)	155 (42.6)	2.14 (1.09-4.17)	0.03
26-35	85 (21.7)	84 (23.1)	1.75 (0.87-3.54)	0.12
36-55	78 (19.9)	85 (23.4)	1.59 (0.79-3.22)	0.20
>55	22 (5.6)	14 (3.8)	2.72 (1.08-6.86)	0.03
**Ethnic group:**				
Malay	235 (60.1)	233 (64.0)	Reference	-
Chinese	49 (12.5)	48 (13.2)	1.01 (0.65-1.57)	0.96
Indian	77 (19.7)	58 (15.9)	1.32 (0.90-1.94)	0.16
Others	30 (7.7)	25 (6.9)	1.19 (0.68-2.09)	0.54
**Monthly income (in Ringgit Malaysia):**				
≤1 k (incl. no income0029	176 (45.0)	173 (47.5)	Reference	-
>1 k	164 (41.9)	156 (42.9)	1.03 (0.76-1.40)	0.83
No information	51 (31.0)	35 (9.6)	1.43 (0.89-2.31)	0.14
**Motorcycle engine capacity (cc):**				
≤200	378 (96.7)	343 (94.2)	Reference	-
>200	5 (1.3)	7 (1.9)	0.65 (0.20-2.06)	0.65
No information	8 (2.0)	14 (3.8)	0.52 (0.22-1.25)	0.52
**Alcohol and illicit substance intake:**				
No	254 (65.0)	297 (81.6)	Reference	-
Yes	61 (15.6)	24 (6.6)	2.97 (1.80-4.91)	<0.001
Unknown	76 (19.4)	43 (11.8)	2.07 (1.37-3.11)	0.001
**Type of road:**				
Intracity	113 (28.9)	105 (28.8)	Reference	-
Highway	100 (25.6)	73 (20.1)	1.27 (0.85-1.90)	0.24
Residential	87 (22.3)	83 (22.8)	1.06 (0.68-1.67)	0.79
Federal road	63 (16.1)	55 (15.1)	0.97 (0.65-1.46)	0.90
Rural	18 (4.6)	31 (8.5)	0.54 (0.29-1.02)	0.60
Others	10 (2.6)	17 (4.7)	0.55 (0.24-1.25)	0.15
**Road configuration:**				
Straight	211 (54.0)	175 (48.1)	Reference	-
Intersection	109 (27.9)	104 (28.6)	0.87 (0.62-1.22)	0.41
Bend	56 (14.3)	65 (17.9)	0.72 (0.47-1.08)	0.11
Others	15 (3.8)	20 (5.5)	0.62 (0.31-1.25)	0.18
**Time of crash (hours):**				
0000-0559	64 (16.4)	46 (12.6)	Reference	-
0600-1159	113 (28.9)	91 (25.0)	0.89 (0.56-1.43)	0.63
1200-1759	109 (27.9)	115 (31.6)	0.68 (0.43-1.08)	0.10
1800-2359	101 (25.8)	104 (28.6)	0.70 (0.44-1.11)	0.13
No information	4 (1.0)	8 (2.2)	0.36 (0.10-1.27)	0.11
**Type of colliding vehicle (first vehicle only):**				
No vehicle	124 (31.7)	112 (30.8)	Reference	-
Two-wheel vehicles	28 (7.2)	27 (7.4)	0.94 (0.52-1.69)	0.83
Four-wheel vehicles	189 (48.3)	209 (57.4)	0.82 (0.59-1.13)	0.22
Larger vehicles	31 (7.9)	6 (1.6)	4.67 (1.88-11.60)	0.001
Unknown	19 (4.9)	10 (2.7)	1.72 (0.77-3.85)	0.19
**Collision type (first collision):**				
Single crash	124 (31.7)	112 (30.8)	Reference	-
Frontal	136 (34.8)	99 (27.2)	1.24 (0.86-1.79)	0.25
Side	71 (18.2)	98 (26.9)	0.54 (0.29-1.00)	0.05
Rear end	19 (4.9)	32 (8.8)	0.65 (0.44-0.97)	0.04
Others	41 (10.5)	23 (6.3)	1.61 (0.91-2.85)	0.10
**Posted speed (km/hr):**				
≤50	32 (8.2)	54 (14.8)	Reference	-
>50	356 (91.0)	309 (84.9)	1.94 (1.22-3.09)	0.05
No information	3 (0.8)	1 (0.3)	5.06 (0.51-50.75)	0.17
**Pre-crash speed (km/hr):**				
≤50	67 (17.1)	116 (31.9)	Reference	-
>50	194 (49.6)	193 (53.0)	1.74 (1.21-2.50)	0.003
No information	130 (33.2)	55 (15.1)	4.09 (2.65-6.33)	<0.001

**Table 2:** Helmet characteristics in cases and controls

**Characteristics**	**Participants, n (%)**			
	**Facial injuries**	**Non-facial injuries**	**Unadjusted OR (95% CI)**	**p-value**
	**(n = 391)**	**(n = 364)**		
**Helmet use:**				
Yes	347 (88.7)	334 (91.8)	Reference	-
No	29 (7.4)	19 (5.2)	1.47 (0.81-2.67)	0.21
Unknown	15 (3.8)	11 (3.0)	1.31 (0.59-2.90)	0.50
**Helmet type:**				
Full-face	6 (1.5)	12 (3.3)	Reference	-
Half-head & open-face	304 (77.7)	293 (80.5)	2.08 (0.77-5.60)	0.15
Tropical	18 (4.6)	21 (5.8)	1.71 (0.54-5.50)	0.36
Not wearing a helmet	29 (7.4)	19 (5.2)	3.50 (0.98-9.53)	0.06
Unknown	34 (8.7)	19 (5.2)	3.58 (1.16-11.07)	0.03
**SIRIM certification:**				
SIRIM certified	264 (67.5)	271 (74.5)	Reference	-
No SIRIM certification	17 (4.3)	22 (6.0)	0.79 (0.41-1.53)	0.49
Not wearing a helmet	29 (7.4)	19 (5.2)	1.60 (1.09-2.36)	0.02
Unknown	81 (20.7)	52 (14.3)	1.57 (0.86-2.86)	0.14
**Type of visor:**				
Integrated	72 (18.4)	93 (25.5)	Reference	-
Added-on	162 (41.4)	190 (52.2)	1.10 (0.76-1.60)	0.61
Helmet without a visor	22 (5.6)	21 (5.8)	1.35 (0.69-2.65)	0.38
Not wearing a helmet	29 (7.4)	19 (5.2)	1.97 (1.02-3.80)	0.04
No information	106 (27.1)	41 (11.3)	3.34 (2.08-5.36)	0.00
**Helmet fixation:**				
Fixed on head	162 (41.4)	220 (60.4)	Reference	-
Came off	105 (26.9)	62 (17.0)	2.30 (1.58-3.34)	<0.001
Not wearing a helmet	29 (7.4)	19 (5.2)	2.07 (1.12-3.83)	0.02
No information	95 (24.3)	63 (17.3)	2.05 (1.40-2.99)	<0.001
*Age of helmet (years):				
≤3	163 (61.7)	176 (56.1)	Reference	-
>3	30 (11.4)	45 (14.3)	0.70 (0.42-1.16)	0.17
Not wearing a helmet	24 (9.1)	19 (6.1)	1.36 (0.72-2.58)	0.34
No information	47 (17.8)	74 (23.6)	0.70 (0.46-1.07)	0.10
*Helmet size:				
55–58 (small-medium)	74 (28.0)	94 (29.9)	Reference	-
59-62 (large-extra large)	164 (62.1)	197 (62.7)	1.06 (0.73-1.53)	0.77
Not wearing a helmet	24 (9.1)	19 (6.1)	1.61 (0.82-3.15)	0.17
No information	2 (0.8)	4 (1.3)	0.64 90.11-3.56)	0.61
*Chin-strap distance:				
0–2 fingerbreadth	185 (70.1)	246 (78.3)	Reference	-
>2 fingerbreadth	28 (10.6)	28 (8.9)	1.33 (0.76-2.32)	0.32
Did not strap	8 (3.0)	5 (1.6)	2.13 (0.69-6.61)	0.19
Not wearing a helmet	24 (9.1)	19 (6.1)	1.68 (0.89-3.16)	0.11
No information	19 (7.2)	16 (5.1)	1.58 (0.79-3.15)	0.20

*non-fatal participants only (n = 264 [cases] *vs* 314 [control]).

A binary logistic regression with a baseline model comprising the helmet type, visor type and helmet fixation as predictors and facial injury as the outcome of interest was constructed for the case control study while the visor type and damage was selected for the helmet laboratory study (sub-set of the case control study). The method used was stepwise backward elimination. Univariate analyses using the Pearson chi square test and Fisher’s exact test were conducted to identify potential confounders. Fisher’s exact test was used in situations where the expected cell frequency was less than five. Selection of potential confounders was based on those variables associated (P < 0.1) with the outcome of interest [26] from the motorcyclist and crash severity independent variables. Confounding factors were tested by adding them into the model consecutively and the factor was retained in the model if the odds ratios of the study factors in the model were changed by more than 10% [27].

Quality of models were assessed using the classification table (sensitivity, specificity and accuracy tests) and this was further emphasised by the Receiver Operating Characteristic (ROC) curve and Hosmer Lemeshow test for goodness of fit. The presence of outliers was assessed using leverage and Cook’s distance and multicollinearity assessment was conducted through the variance inflation factors (VIF).

Missing values were included in the Tables as ‘unknown’ or ‘no information’. The justification to include the missing values into the analyses was based on the nature of the research which involved head injured motorcyclists who were incapable of remembering the event [28]. The unknown status is a common finding in real life situation involving motorcycle-related injuries and this had been reported by various authors [28-31]. ‘Unable to remember event’ or ‘no answer could be obtained’ did not happen randomly and omitting it will produce a biased result [28].

## Results

### Participant characteristics

Of the total 755 study participants, 391 (51.8%) sustained facial injuries. The remaining 364 participants who did not sustain facial injury were used as the control group.

The cases and controls were compared on the range of study factors of interest. Table 1 shows participant and crash characteristics of the cases by facial injury or no facial injury.

#### Demographic characteristics

The majority of injured participants were young male Malay riders of low socio-economic status who rode small motorcycles with engine capacity of 200 cc or less. There were no significant group differences on these variables.

There were no demographic characteristic differences between those sustaining facial injuries and those who did not sustain facial injuries. However, those sustaining facial injuries were also more likely to have reported ingesting alcohol, with or without illegal drugs, prior to the collision compared with the non-facial injury controls (15.6% *vs* 6.6%). The unadjusted odds ratio of alcohol involvement with facial injury was 2.97 (95% Confidence Interval, CI 1.80-4.91, P < 0.001) compared to those who did not consume alcohol with or without drugs.

### Crash characteristics

Facial injury cases were more likely to be involved in a collision on highways and intra-city roads but less likely to be involved in collisions on rural roads, compared with the non-facial injury sample, however none of the road types were shown with statistically significant results in the univariate analysis. Significant group differences were found for type of colliding vehicle, type of collision and pre-crash speed. Collision with a larger vehicle had unadjusted OR of 4.67 (95% CI 1.88-11.60, P < 0.001) for sustaining facial injury. Among all collision types, only the rear-end collision showed statistically significant results (P = 0.04) with unadjusted OR of 0.65 (95% CI 0.44-0.97). Pre-crash speed of more than 50 km/hr had slightly higher unadjusted OR [1.74 (95% CI 1.21-2.50)] compared to speed of less than 50 km/hr for sustaining facial injuries.

#### Helmet characteristics

Table 2 shows helmet characteristics by group. The majority of participants wore a helmet during the crash, with the non-facial injury group were shown with higher percentage compared with the facial injury group (91.8% *vs* 88.7%). In general, majority wore the half-head and open face (77.7% among the facial injury group and 80.5% among the non-facial injury group), followed by the tropical (4.6% and 5.8%) and the full-face helmets (1.5% and 3.3%). The distribution of the unhelmeted users were 7.4% (facial injury group) and 5.2% (non-facial injury group).

Unhelmeted motorcyclists were shown to have statistically significant or near significant associations in helmet type, SIRIM certification, type of visor and helmet fixation with facial injury and the unadjusted OR in all these categories were shown as 3.50 (95% CI 0.98-9.53), 1.60 (95% CI 1.09-2.36), 1.97 (95% CI 1.02-3.80) and 2.07 (95% CI 1.12-3.83) respectively. Participants with dislodged helmets had stronger association with higher unadjusted OR, 2.30 (95% CI 1.58-3.34, P < 0.001) compared to unhelmeted motorcyclists for sustaining facial injury.

Non-fatal cases (n = 578) were also requested to report on the fixation status of their helmets, measured by chinstrap distance. Overall, the majority of participants reported firmly fastening their helmets, however, the non-facial injury group were more likely to report doing so, compared with the facial injury group (78.3% *vs* 70.1%). Those who did not strap their helmets at all had higher unadjusted OR 2.13 995% CI 0.69-6.61) of having facial injury compared to those who fastened their helmets loosely [unadjusted OR of 1.33 (95% CI 0.76-2.32)] and unhelmeted motorcyclists [unadjusted OR 1.68 (95% CI 0.89-3.16)], however all associations were shown to be statistically not significant.

### Quantification of helmet type, visor type and helmet fixation with facial injuries

The association of helmet and visor type and helmet fixation on facial injuries was examined using the sub-set of the sample who sustained a facial injury, adjusting for confounding factors. A total of 676 facial injuries were sustained, 177 (26.2%) were upper face injury, 295 (43.6%) middle face injury and 204 (30.2%) lower face injury. Table 3 shows the outcomes of logistic regression analyses examining facial injury outcomes by helmet variables.

**Table 3:** The effect of helmet fixation, helmet type and visor type with facial injury and its divisions

	*Facial injury	**Upper face injury	^**#**^**Middle face injury**	^**##**^**Lower face injury**
**Explanatory variables**	**AOR (95%CI)**	**AOR (95%CI)**	**AOR (95%CI)**	**AOR (95%CI)**
**Helmet fixation**				
Fixed	1.00	1.00	1.00	1.00
Not fixed	2.10 (1.41-3.13)	6.64 (3.71-11.91)	5.36 (3.05-9.44)	2.00 (1.22-3.26)
Unknown	1.98 (1.30-3.04)	4.16 (2.17-7.98)	2.63 (1.34-5.17)	1.80 (1.03-3.14)
**Type of helmet**				
Full-face	1.00	1.00	1.00	1.00
Half-head & open- face	1.43 (0.50-4.04)	1.01 (0.23-4.46)	0.52 (0.13-2.16)	4.38 (0.54-35.80)
Tropical	1.47 (0.43-5.10)	1.53 (0.25-9.27)	1.17 (0.20-6.68)	5.51 (0.58-52.14)
Not wearing a helmet	3.20 (1.01-10.16)	7.34 (1.34-40.30)	1.75 (0.37-8.19)	13.28 (1.51-116.54)
Unknown helmet	0.55 (0.15-2.07)	0.29 (0.05-1.73)	0.26 (0.05-1.46)	1.74 (0.17-17.58)
**Type of visor**				
Integrated	1.00	1.00	1.00	1.00
Added-on	0.91 (0.60-1.37)	0.57 (0.30-1.11)	0.55 (0.28-1.09)	0.91 (0.55-1.53)
Helmet without a visor	0.90 (0.44-1.84)	0.76 (0.25-2.33)	0.45 (0.14-1.43)	0.70 (0.27-1.83)
Unknown	2.79 (1.48-5.28)	5.95 (2.47-14.33)	4.12 (1.90-8.93)	2.77 (1.31-5.86)

*Controlled for alcohol and illegal substance use, type of colliding vehicle.

**Controlled for age, alcohol and illegal substance use, type of colliding vehicle and type of collision.

^#^Controlled for alcohol and illegal substance use, type of colliding vehicle.

^##^Controlled for riding position, alcohol and illegal substance, type of colliding vehicle and type of collision.

Among all the explanatory variables, helmet fixation showed consistent significant effects with all facial injury location outcomes. The findings showed that, overall, participants with non-fixed helmets were adjusted odds ratio(AOR) of 2.10 (95% CI 1.41-3.13) to sustain a facial injury, and 6.64 (95% CI 3.71-11.91), 5.36 (95% CI 3.05-9.44) and 2.00 (95% CI 1.22-3.26) to sustain an upper face injury, middle face injury and lower face injury, respectively, compared with participants who wore fixed helmets.

In addition, some associations between facial injury and helmet type were found. Unhelmeted motorcyclists showed significant associations with upper face and lower face injuries. This group had AOR of 7.34 (95% CI 1.34-40.30) and 13.28 (95% CI 1.51-116.54) to sustain an upper face and lower face injury compared with participants who wore a full-face helmet.

The users of the added-on visors and helmets without a visor had less odds of sustaining facial injury in all divisions compared to the integrated visor users, however most of the associations were statistically not significant.

### Analysis of helmet components

Analysis of visor components and damage was undertaken to assess the relationships between visor type, visor damage and facial injury. In total, 140 visors were obtained (five helmets were visorless). Of the 140 visors, only 103 (73.6%) were available for examination. Almost half (49.7%) were the integrated type while 46.9% were the added-on type. A high proportion, (46.2%) were not SIRIM certified. Both the integrated and added-on visors were made of polycarbonate material and had similar thickness between 1.84 to 2.07 mm.

Table 4 presents the outcome of the final logistic regression model regarding the effect of visor type and damage on facial injury. The visor type showed similar results in both the case control study as well as in the laboratory component study where the added-on visors had a lower odds of being associated with sustaining facial injuries (adjusted odds ratio (AOR) = 0.91, 95% CI 0.60-1.37) in the case control study and AOR = 0.50 (95% CI 0.24-1.05) in the helmet laboratory study, compared to the integrated visors.

**Table 4:** The effect of visor type and visor damage with facial injury and its divisions

	^*^Facial injury	**Upper face injury	^**#**^**Middle face injury**	^**##**^**Lower face injury**
**Type of visor**				
Integrated	1.00	1.00	1.00	1.00
Added-on	0.50 (0.24-1.05)	0.67 (0.24-1.92)	0.44 (0.21-0.92)	0.53 (0.24-1.14)
Helmet without a visor	0.89 (0.06-12.27)	-	3.60 (0.33-39.24)	-
**Visor damage**				
No damage	1.00	1.00	1.00	1.00
With damage	5.48 (1.46-20.57)	-	4.51 (1.08-18.86)	7.51 (0.91-62.34)

*Controlled for type of colliding vehicle.

**Controlled for type of colliding vehicle.

^#^Controlled for type of collision.

^##^Controlled for ethnic group.

Generally, all components investigated did not reveal any statistically significant associations with facial injury, except for visor damage. A damaged visor was associated with a higher odds of facial injury (AOR = 5.48, 95% CI 1.40-20.57; P = 0.01), middle face injury (AOR = 4.51, 95% CI 1.08-18.86; P = 0.04), and lower face injury (AOR = 7.51, 95% CI 0.91-62.34; P = 0.06).

Almost one-third (31.7%) of the visors were categorised as structural damage. Of these, 55.9% had crash marks only and 9.0% were without damage. The damage was assessed by two observers based on ‘yes, there is damage’ and ‘no, there is no damage’. Old crash marks from previous crash was assigned as ‘no damage’. Kappa statistic revealed good agreement on the extent and type of visor damage (κ = 0.94; P < 0.001).

## Discussion

Three causative factors for motorcycle-related facial injury were examined in this research: helmet wearing, helmet fixation status and visor damage. Study findings clearly demonstrated that wearing a helmet significantly reduced risk of facial injury. More importantly, it has highlighted the importance of helmet fixation and suggests that helmet fixation is a stronger predictor in determining facial injury than helmet type. This study is, to our knowledge, the first to examine the effects of helmet type, fixation and visor damage on facial injury and show the substantial beneficial effects of helmet fixation for protection against facial injury, and the odds associated with visor damage.

Helmet fixation has long been identified as one of the grey areas in motorcycle helmet research [32]. While the effect of helmet dislodgement was identified over thirty years ago as contributing to serious and fatal head injuries amongst motorcyclists [33], there has been little unequivocal evidence attesting to this since. Moreover, there has been no evidence reported on the association of dislodgement with facial injury. The current study provides clear evidence that one of the primary determinants of helmet dislodgment or displacement is failure to fasten the helmet properly.

In addition, our findings showed that wearing any helmet has a protective effect on the risk of facial injury. Hurt and colleagues (1981) showed that helmet protectiveness depended on the area of coverage, suggesting that the larger the area, the more effective the helmet and argued that a full-face helmet is superior as it provides more facial coverage than other helmets especially to the lower face [34]. Further, the design of the full-face helmet itself prevents it from being dislodged easily. Among all the divisions of facial injury, the upper face injury was more profoundly affected by helmet dislodgment compared to other facial divisions. Overall, the most severe form of facial injuries in relation to helmet type were shown as below:

i) Full-face helmets: mid-facial fractures (AIS 2)

ii) Half-head and open-face: pan-facial fractures or Le Fort 3 fractures (AIS 3)

iii) Tropical helmet: pan-facial fractures (AIS 3)

A key interest was the effect of visor damage on sustaining a facial injury. Motorcycle helmet visors are not designed for impact absorption and distribution. The thin polycarbonate shield is generally provided to protect the face from dust, road fragments and other small particles. There is scant literature addressing the issues of visor properties related to facial injuries. To date, only one study [34] investigated the effect of motorcycle helmet visor in providing protection to the face particularly to the eye however, concluded that helmet design was the protective factor, and not the visor.

A visor, when subjected to an impact, will bend and break depending on the magnitude and direction of the force. The phenomenon of bending or/and breaking could cause injury to the face, and it might be expected that visor damage and risk of injury is more likely with added-on visors compared with integrated visors. While this was examined in this study, no significant additional risk associated with added-on visors was found. Notwithstanding, it is important to note that, in this study, the most common type of facial injury observed was the abrasion wound. A laceration was also a common injury and was shown to be associated with a shattered visor as well as with some structurally intact visor. Further, added-on visors are quite popular among Malaysian motorcyclists, with 41.4% of participants with facial injury and 54% of participants with non-facial injury participants were wearing this type of visor during the crashes. Most of these visors were not certified by SIRIM.

### Limitations

The main limitation of this research was missing data especially on helmet type and fixation status, which was most prevalent in fatal cases and those with concomitant head injury as well as fatal cases. As highlighted earlier, missing data and unknown helmet status is common in motorcycle injuries studies [28-31,35] and may cause a biased result if these cases are omitted from the analyses [28].

Apart from missing data and information recall, potential bias in this study was also related to participant selection. Participants were recruited at the Emergency Departments (ED) and selected from a large number of patients with all range of injuries. High refusal rate was encountered among the mildly injured participants because of long waiting times (patients were approached only after they have completed treatment at the ED). In addition, patients with mild to moderate injuries were commonly treated as outpatients and were therefore likely to visit private out-patient clinics. Recruitment of out-patient participants was not carried out at these settings for logistic reasons.

Delay in fatal report completion was another limitation in this study. The issue of incomplete records and delays in finalizing reports is well-known among the enforcement officers. The delay was mainly related to the autopsy report. Fatal cases with incomplete reports were omitted from the study and this contributed substantially to the reduction of the sample size.

Crash severity is an important potential confounding factor in this study. The established standard of determining crash severity is by performing in-depth crash investigation. However, the intensity of effort required to complete one in-depth crash investigation makes this prohibitively resource intensive. Due to this circumstance, we used a self report approach to estimating crash severity that is widely used in the literature [36-38].

## Conclusions and policy implications

A helmet is effective for protection against head and facial injuries provided it is worn properly. The effect of improper use and fixation status had been shown to increase the risk of facial injuries. The findings of this study have provided a major step forward in our understanding of the effectiveness of helmet wearing behaviour on reducing facial injuries.

The implications of these findings are substantial and can be used to guide the development of strategies and initiatives to improve helmet wearing behaviour in Malaysia. Moreover, the findings of this study could be extrapolated to motorcyclist safety in other countries such as Indonesia, China, Vietnam and India which reported similar helmet wearing issues [20-24].

Injury prevention programmes targeting improper helmet use and fit, have the potential to result in a significant reduction in injuries among motorcyclists in Malaysia. The current Malaysia Helmet Law Motorcycle (Safety Helmets) (Amendment) Rules 2010 state that:

‘Every person, other than a person exempted under rule 5, who drives or rides on a motor-cycle on a road shall wear a safety helmet on his head fitted and securely fastened in the manner required by the nature and construction of the safety helmet. Any person who contravenes this rule shall be guilty of an offence’.

However, to date, legal actions are only taken to motorcyclists who fail to wear a helmet. Motorcyclists who wear improperly secured helmet only receive verbal warning and advice from the police [39]. To achieve greater reductions in head and facial injuries amongst motorcyclists, recommendations for policy, enforcement strategies and improved helmet design are suggested, including revisions to helmet laws to include specific legislation on fixation status, enhanced enforcement programmes, educational and behavioural modification initiatives.

Another significant finding in this study was the effect of visor design and damage on facial injury. Again, no study within this geographical region (with similar visor design) has reported patterns of facial injury associated with the visor.

Rule 3 [2] of the same Road Transport Act states that:

‘the safety helmet may be fixed with a clear colourless visor of any kind provided that any part, extension or attachment thereof shall not conceal or obscure any portion of the face between the eyebrow and the chin’.

The finished end surface or the edge of a visor has been shown to be a critical factor in the occurrence of facial injury. New laboratory test should be included to test the ‘cutting potential’ of the visor edge. This could be included into the Malaysian Standard MS1-2:2011 which currently only addressed the consequences of a shattered visor (40).

## Abbreviations

ASEAN: Association of Southeast Asian Nations; SIRIM: Standards and Industrial Research Institute of Malaysia; CI: Confidence interval; FTIR: Fourier transform infrared; AIS: Abbreviated injury scale; AOR: Adjusted odds ratio; ED: Emergency Departments.

## Competing interests

There were no competing interests during the conduct of this study.

## Authors’ contributions

RR and RM designed the study, guided data collection and designed the analysis and revised the paper. RR designed the data collection tool, collected data for the whole study and trained the contracted staff who collected data in one hospital, analyzed the data, drafted and revised the paper. JO contributed to all aspects of the design and conduct of the study, guided data collection and revised the draft. PH and AFS contributed to specific aspects of the design and conduct of the study in accordance with their specialist expertise and contributed to the review and revisions of the paper. All authors read and approved the final manuscript.

## Pre-publication history

The pre-publication history for this paper can be accessed here:

http://www.biomedcentral.com/1471-227X/14/17/prepub
